# Hypoallergenic and Low-Protein Ready-to-Feed (RTF) Infant Formula by High Pressure Pasteurization: A Novel Product

**DOI:** 10.3390/foods8090408

**Published:** 2019-09-12

**Authors:** Md Abdul Wazed, Mohammed Farid

**Affiliations:** Department of Chemical and Materials Engineering, University of Auckland, Private Bag 92019, Auckland 1142, New Zealand; mwaz610@aucklanduni.ac.nz

**Keywords:** alpha-lactalbumin (α-Lac), beta-lactoglobulin (β-Lg), high pressure processing (HPP), pasteurization, ready-to-feed (RTF) infant formula

## Abstract

Infant milk formula (IMF) is designed to mimic the composition of human milk (9–11 g protein/L); however, the standard protein content of IMF (15 g/L) is still a matter of controversy. In contrast to breastfed infants, excessive protein in IMF is associated with overweight and symptoms of metabolic syndrome in formula-fed infants. Moreover, the beta-lactoglobulin (β-Lg) content in cow milk is 3–4 g/L, whereas it is not present in human milk. It is considered to be a major reason for cow milk allergy in infants. In this respect, to modify protein composition, increasing the ratio of alpha-lactalbumin (α-Lac) to β-Lg would be a pragmatic approach to develop a hypoallergenic IMF with low protein content. Such a formula would ensure the necessary balance of essential amino acids, as 123 and 162 amino acid residues are available in α-Lac and β-Lg, respectively. Hence, in this study, a pasteurized form of hypoallergenic and low-protein ready-to-feed (RTF) formula, a new product, is developed to retain heat-sensitive bioactives and other components. Therefore, the effects of high pressure processing (HPP) under 300–600 MPa at approximately 20–40 °C and HTST pasteurization (72 °C for 15 and 30 s) were investigated and compared. The highest ratio of α-Lac to β-Lg was achieved after HPP (600 MPa for 5 min applied at 40.4 °C), which potentially explains the synergistic effect of HPP and heat on substantial denaturation of β-Lg, with significant retention of α-Lac in reconstituted IMF. *Industrial relevance*: This investigation showed the potential production of a pasteurized RTF formula, a niche product, with a reduced amount of allergenic β-Lg.

## 1. Introduction

Infant milk formula (IMF) is intended to serve as a functional substitute for infants under 12 months of age. The World Health Organization (WHO) recommends absolute breastfeeding of infants for the first 6 months of age [1], whereas the American Society of Paediatrics suggests the same for at least 12 months [2]. However, only 38% of infants are being breastfed globally [3], which indicates the common use of IMF, a $41 billion USD market [4]. IMF is available in three forms—powdered, liquid concentrate, and liquid ready-to-feed (RTF). Among them, RTF, the most convenient form, is currently manufactured as a sterilized product to ensure safety using UHT (Ultra High Temperature, 135–145 °C for a short time). However, UHT causes the nutritional profile of RTF formula to deteriorate, especially vitamin A, B, and D, along with protein denaturation, which requires supplementation of micronutrients (e.g., vitamins and minerals) [5]. Although every possible effort is being made to bring IMF closer to human milk (HM), there is still a gap between them nutritionally, which governs neurological, physiological, and immunological growth and development of infants [6,7,8]. Such a difference could be explained by the nutritional balance that remains fixed in an IMF, while it varies in HM throughout the lactation period and even between individuals [9].

Moreover, beta-lactoglobulin (β-Lg) represents about 50% of total whey as a major whey protein in cow milk. It is also considered a major allergen to infants despite having numerous functional and nutritional roles in adult human health. However, interestingly, this β-Lg is absent in HM. To address the cow’s milk allergy (CMA), researchers and manufacturers introduced partially hydrolyzed formula (pHF) and extensively hydrolyzed formula (eHF), which are also recommended by paediatricians to reduce early allergy manifestation [10]. In pHF and eHF, enzymatic hydrolysis of proteins with digestive enzymes reduces the allergic properties by breaking them into small peptides and free amino acids, which are not allergenic [11]. However, these hydrolyzed formulas often exhibit bitter taste, poor flavour, reduced lipid emulsifiability, and elevated osmolality, which limit their application in general IMF [12].

On the other hand, alpha lactalbumin (α-Lac) is a bioactive protein present in all mammal milk, which is regarded as a component of lactose synthesis with antimicrobial, prebiotic, and Ca-binding capacity [13]. The α-lac content in human milk is 3–4 g/L, while it is only 1 g/L in mature cow milk. It contains a high level of different essential amino acids like tryptophan, lysine, and cysteine, and hence, fortification of IMF with α-Lac is recommended [13,14].

The standard protein content of infant formula is still a matter of controversy since formula production aims to mimic HM [14]. HM contains 9–11 g/L protein [15], while conventional infant formula provides 15 g/L [16]. Burgeoning demand for low-protein infant formula, especially in Asia, resulted mainly from paediatric obesity [17]. Moreover, excess protein also induces unnecessary strain on immature metabolic organs [18,19]. Thus, the possible alternative could be reducing the protein content and adding free amino acid into infant formula, which would be unphysiological since metabolic consequences of free amino acids are mostly unknown [14]. Thereupon, a logical approach would be to modify the protein profile of formula to make it closer to HM by increasing the ratio of α-Lac to β-Lg, which will yield a formula that has lower total protein, but retains the necessary balance of essential amino acids [14,16,20,21]. Moreover, this would also be an imperative strategy because complete removal of allergenic β-Lg would deteriorate the function of milk proteins since it is necessary for whey proteins to be associated with casein micelles [22]. High pressure processing (HPP) defragments casein micelles into smaller particles and splits them into more soluble components like αs1-, αs2-, β-, and k-caseins [23,24,25]. More than a 50% reduction in the size of casein micelle was observed after HPP at >300 MPa at 40 °C [24]. In powdered IMF, reducing the protein content to as low as 9.77 g protein/100 g did not cause a significant impact on physical stability and shelf-life [26]. However, in RTF liquid IMF, a higher ratio of α-Lac to β-Lg reduces the viscosity and induces rapid sedimentation during storage, as observed in UHT-treated products; and hence, the addition of thickener and stabilizer is suggested to manage the viscosity and sedimentation, respectively [27,28,29]. Furthermore, Crowley et al. [27] reported increased α-Lac reduced heat-induced coagulation in a model whey protein-dominant IMF. They further observed lower protein–protein interactions in the model IMF due to the fortification of IMF with α-Lac. Additionally, Kamarei [30] patented his invention in manufacturing refrigeration-shelf-stable pasteurized IMF with required quantities of nutrients. In this patent, he reported that UHT-treated RTF formula provides a different and unknown amount of degradable micro nutrients due to the high-heat treatment and subsequent storage, which also affects the nutritional value and sensory attributes. These outcomes evidence the possibility of developing an IMF with a higher ratio of α-Lac to β-Lg.

Previously, Huppertz et al. [31] described the mechanism of HPP-induced denaturation of α-Lac and β-Lg at 200–800 MPa/20 °C in whole milk and reported α-Lac as a pressure-resistant protein, unlike β-Lg. Furthermore, Mazri et al. [32] also investigated the denaturation kinetics of these two bioactive proteins in skim milk under HPP of 450–700 MPa at 20 °C and confirmed the baroresistance of α-Lac in comparison to β-Lg. Recently, HPP has been recommended to preserve human milk, due to its efficient inactivation of microbial pathogens, along with the retention of unique components [33]. Wesolowska et al. [34] also reviewed the effect of HPP to pasteurize human milk and referred to HPP as superior to the conventional holder pasteurization (63 °C/30 min) in maintaining bioactivity of protein components. To the best of our knowledge, the synergistic effect of HPP and heat to achieve a higher ratio of α-Lac to β-Lg has not been explored in IMF, which fundamentally pioneered this work.

Therefore, the aim of this work was to achieve a higher ratio of α-Lac to β-Lg through investigating their retention after HPP at different pressure–temperature–time combinations in reconstituted IMF, fortified with α-Lac. For comparison, we also performed high-temperature short-time (HTST) pasteurization at 72 °C for 15 and 30 s, and thereafter measured the concentration of α-Lac and β-Lg. The combined effect of pressure and temperature on the kinetics of denaturation of both proteins was also analyzed.

## 2. Materials and Methods

### 2.1. α-Lac-Added Reconstituted IMF Preparation

Bovine α-Lac powder (native form) was generously donated by Davisco Foods International, Le Sueur, MN, USA. The protein content and α-Lac level in the powder were more than 95% and 90%, respectively. Spray-dried IMF powder (stage 1, 1.4 g protein/100 mL, whey protein to casein ratio 60:40) was reconstituted into cooled, boiled milli-Q water, as per the instructions of the manufacturer. Finally, 100 mg of α-Lac was added into 100 mL of reconstituted IMF, and a magnetic stirrer was used for gentle mixing.

### 2.2. HTST Treatment

HTST treatments at 72 °C for 15 and 30 s were performed in duplicate, using a sample (15 mL) in a copper tube, then immersing it in a water bath (72.2 °C). A thermocouple (K-type) was inserted at the geometric centre of the copper tube (29 cm length, 9.6 mm outer diameter, and 8.0 mm inner diameter) to record the temperature–time profile using a data logger. The sample temperature increased sharply within 30 s to achieve the steady-state temperature. The sample tube was immediately transferred to an ice bath after treatment. It is to be noted that the determination of α-Lac and β-Lg content following ELISA (Section 2.4) requires a large amount of sample (15 mL). For this reason, this study could not follow the capillary method to perform HTST treatments.

### 2.3. HPP Treatment

A QFP 2L-700 HPP unit (Avure Technologies, Columbus, OH, USA) was used to perform the combined pressure–heat treatments. The equipment has a 2 L cylindrical stainless steel pressure treatment chamber with two internal thermocouples for monitoring the temperature. The system also includes a heating system, water circulation, and a pumping system, along with a computer-operated control system. Distilled water was used as a pressure transmission fluid, and isostatic pressure transmission resulted in a uniform pressure in all directions [35].

In HPP treatments, vacuum-packaged 15 mL samples were placed into the treatment chamber and immediately cooled in an ice water bath after treatment. HPP of 300 MPa (10 and 20 min), 400 MPa (10 and 20 min), 500 MPa (5 and 10 min), and 600 MPa (1 and 5 min) were applied at different temperatures, ranging from ambient temperature to ~40 °C. The treatment time refers to the duration of steady-state pressure conditions, as programmed in the operating system. The temperature increase was about 2.3 °C/100 MPa, due to the adiabatic heating during pressurization, and the decompression time was <20 s in all treatments.

**Figure 1:**
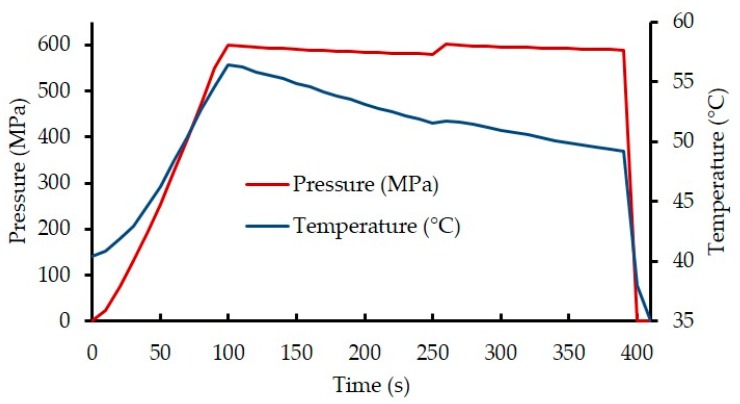
Temperature-pressure profile in alpha-lactalbumin (α-Lac)-added reconstituted infant milk formula (IMF): High pressure processing (HPP) applied at 40.4 °C/600 MPa for 5 min.

However, similar to Evelyn and Silva [36], the temperature of the HPP chamber dropped steadily during the steady-state pressure phase of the HPP cycle, due to cooling followed by a rapid drop in temperature during decompression (Figure 1).

Transient temperature change significantly impacts all chemical and biological reactions, due to its exponential effect on them. The average temperature during the steady-state pressure phase does not represent the accurate representation of the treatment temperature. For instance, as shown in Figure 1, the pressure come-up time (100 s) prior to achieving the steady-state pressure condition is relatively long and must be accounted for. Considering this, Farid and Alkhafaji [37] provided an integrated value of processing temperature, named “effective treatment temperature (*T*_eff_)”, to represent the entire treatment period (Equation (1)).

(1) $$T_{\mathrm{eff}}=\frac{-E/R}{\ln(\sum_{0}^{n}e^{-E/RT}/n-1)}$$

where *T* (K) is the absolute temperature recorded at a given time interval (t), *E* is the activation energy (kJ/mol) of the specific biological or chemical reaction, *R* is the gas constant (8.314 J/mol/K), and *n* is the number of temperature recording points measured at equal time intervals.

From now on, the temperature in HPP treatments referred to in this paper will be the *T*_eff_.

### 2.4. Sample Preparation for α-Lac and β-Lg Determination

Defatted whey supernatant was prepared from both HTST- and HPP-treated samples by centrifuging them at 9000 g for 15 min at 4 °C. Then, the whey portion was obtained by adjusting the pH to 4.6 using 8 M acetic acid, followed by centrifugation at the same conditions. The pH of the supernatant was readjusted to 6.8 using 3 M NaOH.

### 2.5. Determination of α-Lac and β-Lg Content

An enzyme-linked immunosorbent assay (ELISA) method was followed to measure the concentration of α-Lac and β-Lg. The sandwich ELISA was performed using bovine α-Lac and β-Lg Quantitation Kits (catalogue E10-128 and E10-125, respectively) and an ELISA Starter Accessory Kit (catalogue E103), purchased from Bethyl Laboratories, TX, USA. The ELISA plates were coated, and supplied bovine α-Lac and β-Lg were diluted to obtain standard curves, following the manufacturer’s protocols. A multimode plate reader with associated software (Perkin Elmer’s EnSpire Multimode Plate reader, Waltham, Massachusetts, USA) was used to read the absorbance of ELISA plates at 450 nm. The unknown concentration of α-Lac and β-Lg of treated samples was obtained in duplicate from the standard curves. Retention of α-Lac and β-Lg was calculated as a percentage of α-Lac and β-Lg in untreated samples, respectively, as given in Equation (2).

(2) $$\mathrm{Retention}=\frac{c_{t}}{c_{o}}\times 100,$$

where *c*_t_ and *c*_o_ represent the concentrations of α-Lac or β-Lg after and before treatment, respectively.

### 2.6. Kinetics of Protein Denaturation

Activation energy (*E*a) for α-Lac and β-Lg denaturation was calculated using the results obtained from HPP-treated samples. The denaturation of α-Lac and β-Lg with time after HPP treatment can be described by the general rate equation

(3) $$-\frac{dc}{dt}=kc^{n},$$

where $-\frac{dc}{dt}$ is the rate of denaturation, *k* is the rate constant, *c* is the concentration of α-Lac or β-Lg, and *n* is the order of reaction.

It has been reported that HPP denaturation of α-Lac follows first-order kinetics in whole milk [38]. For first-order kinetics (*n* = 1), the integration of Equation (3) gives

(4) $$\ln\left(c_{t}/c_{o}\right)=-kt.$$

The semi-logarithmic plot of Equation (4) gives a straight line with high coefficients of correlation (*r*^2^), and the value of the ordinate intercept *b* (time, *t* = 0) appears close to zero. The slopes of the lines obtained correspond to the rate constant (*k*).

Furthermore, the reaction kinetics for β-Lg in HPP was also reported as a second-order reaction by Anema et al. [39] in skim milk and Hinrichs et al. [40] in whey proteins. For non-first order kinetics (*n* ≠ 1), the integration of Equation (3) gives

(5) $${\left(c_{t}/c_{o}\right)}^{1-n}=1+\left(n-1\right)kt.$$

The graphical representation of Equation (5) yields straight lines, and the ordinate intercept b (time, *t* = 0) should be 1 if the treatment follows the estimated reaction order. The rate constant (*k*) is obtained from the slope of the lines. The Arrhenius equation relates the treatment temperature and the rate constant of a denaturation process as given in Equation (6):

(6) $$k=Ae^{-E_{a}/RT},$$

where *A* is the pre-exponential factor, *E*_a_ is the activation energy, *R* is the universal gas constant, and *T* is the absolute temperature.

By taking the logarithms of both sides, Equation (6) gives a linearized form as

(7) lnk=(−Ea/R)×(1/T)+lnA.

The graphical representation of Equation (7) (lnk vs. 1/*T*) determines the effect of temperature on the rate of constant (*k*). The gradient of Equation (7) is equal to –*E*a/R, and thus the activation energy (*E*a) is calculated.

### 2.7. Statistical Analysis

Statistical analysis of data, including mean and standard deviation for replicates, was performed using Microsoft Excel 2013 (Microsoft Inc., Redmond, Washington, USA). The level of significance was set at *p* = 0.05.

## 3. Results and Discussion

### 3.1. Calculation of Effective Treatment Temperature (T*_eff_*)

In this study, *T*_eff_ for HPP treatments on α-Lac and β-Lg was calculated using Equation (1) and tabulated in Table 1. In all cases, *T*_eff_ was significantly lower than the maximum temperature in HPP (*T*_max_), which further establishes the application of the concept of *T*_eff_ in denaturation studies.

### 3.2. Kinetics of α-Lac and β-Lg Denaturation during HPP Treatments

**Figure 2:**
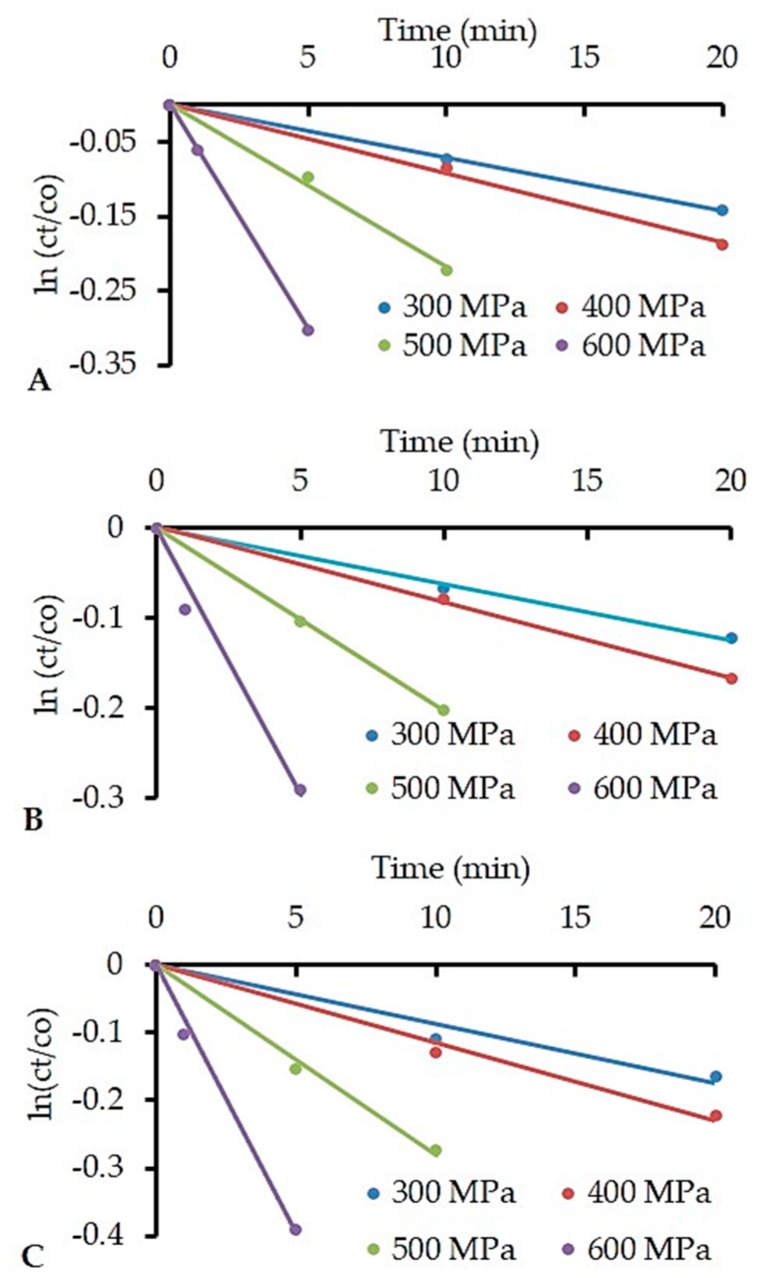
High-pressure denaturation of alpha-lactalbumin (α-Lac), HPP applied at ~20 °C (**A**), ~30 °C (**B**), and ~40 °C (**C**). Temperature at all HPP conditions (*T*_eff_) is presented in Table 1. The concentration of α-Lac is expressed as *c*_t_/*c*_o_, where *c*_t_ = α-Lac concentration after HPP and *c*_o_ = initial α-Lac concentration before HPP.

**Figure 3:**
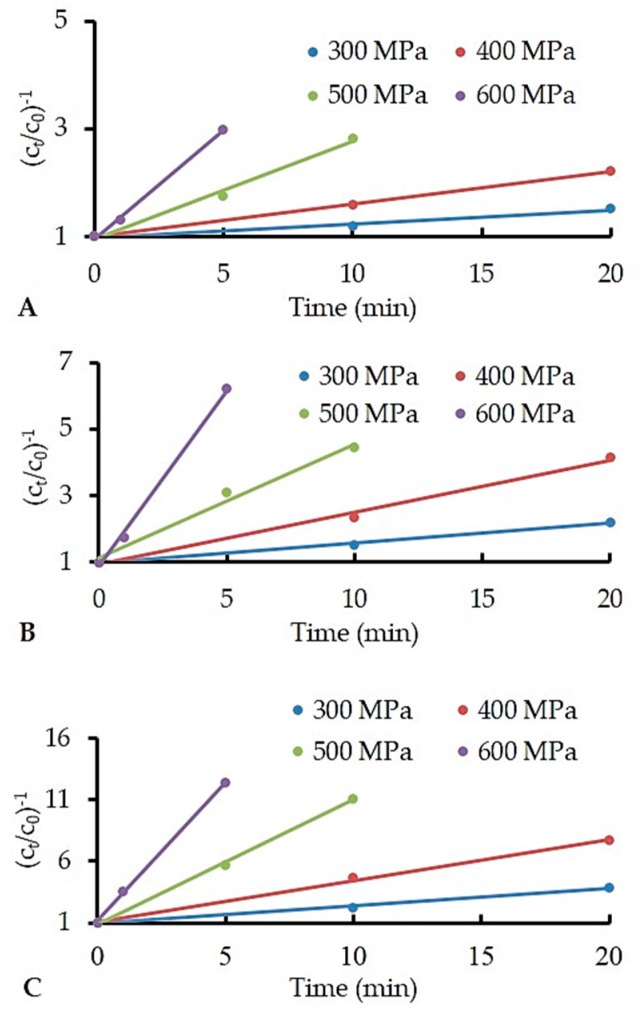
High-pressure denaturation of beta-lactoglobulin (β-Lg), HPP applied at ~20 °C (**A**), ~30 °C (**B**), and ~40 °C (**C**). Temperature at all HPP conditions (*T_eff_*) is presented in Table 1. The concentration of β-Lg is expressed as *c*_t_/*c*_o_, where *c*_t_= β-Lg concentration after HPP and *c*_o_ = initial β-Lg concentration before HPP.

Activation energy (*E*a) for α-Lac and β-Lg denaturation was calculated using the results obtained from HPP treated samples following Section 2.6. The reaction orders for HPP-induced denaturation of α-Lac and β-Lg were determined to compare the rate constants (*k*) at different temperatures and pressures, and to calculate the *E*a. Experimental data obtained in this work and their graphical representation in Figure 2 and Figure 3 yielded the reaction order (*n*) as 1 and 2 for α-Lac and β-Lg, respectively. These orders consistently produced reasonably straight lines with good correlation of coefficients (*r*^2^ > 0.98) and agree well with previous studies [38,39,40].

**Figure 4:**
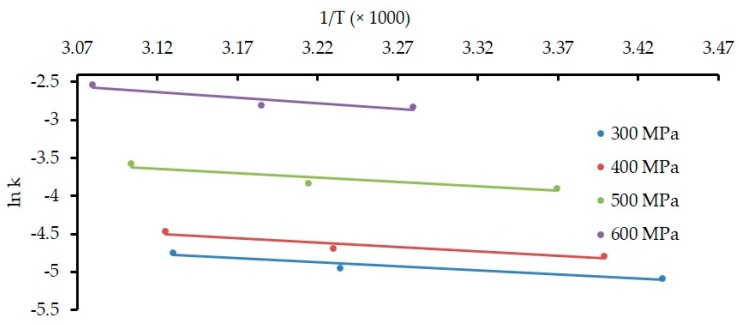
Effect of HPP on the rate of constant (*k*) for denaturation of α-Lac. Temperature at all HPP conditions (*T*_eff_) is presented in Table 1.

**Figure 5:**
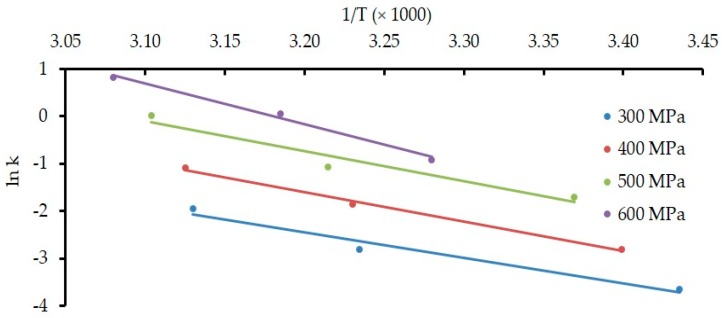
Effect of HPP on the rate of constant (*k*) for denaturation of β-Lg. Temperature at all HPP conditions (*T*_eff_) is presented in Table 1.

Furthermore, Figure 4 and Figure 5 represent the effect of HPP on the rate constant (*k*) for the denaturation of α-Lac and β-Lg, respectively. $E_{a}$ was calculated from the gradients of respective lines from Figure 4 for α-Lac and from Figure 5 for β-Lg. *E*_a_ indicates the energy barrier that a protein is required to overcome to take part in a reaction. Values of $E_{a}$ during HPP are presented in Table 2 where we observed a distinctive higher *E*_a_ in β-Lg denaturation compared to α-Lac. These results correspond well with the previous studies investigated by Huppertz et al. [31] for whole milk and Mazri et al. [32] for skim milk.

However, *E*_a_ values obtained in this work for β-Lg denaturation differ to some extent from those reported earlier by Anema et al. [39] from HPP treatments of 200–600 MPa at 10–40 °C up to 60 min. They reported *E*_a_ as 103.8 and 114.35 kJ/mol for 500 and 600 MPa, respectively; twice higher than the reported values of this work.

IMF is a complex food containing a variety of ingredients. Therefore, this difference in *E*_a_ could be attributed to the dissimilarity in treatment media, treatment duration, and the estimation of treatment temperature, since this study considered the temperature attained during the pressure come-up time.

### 3.3. Ratio of α-Lac to β-Lg

**Figure 6:**
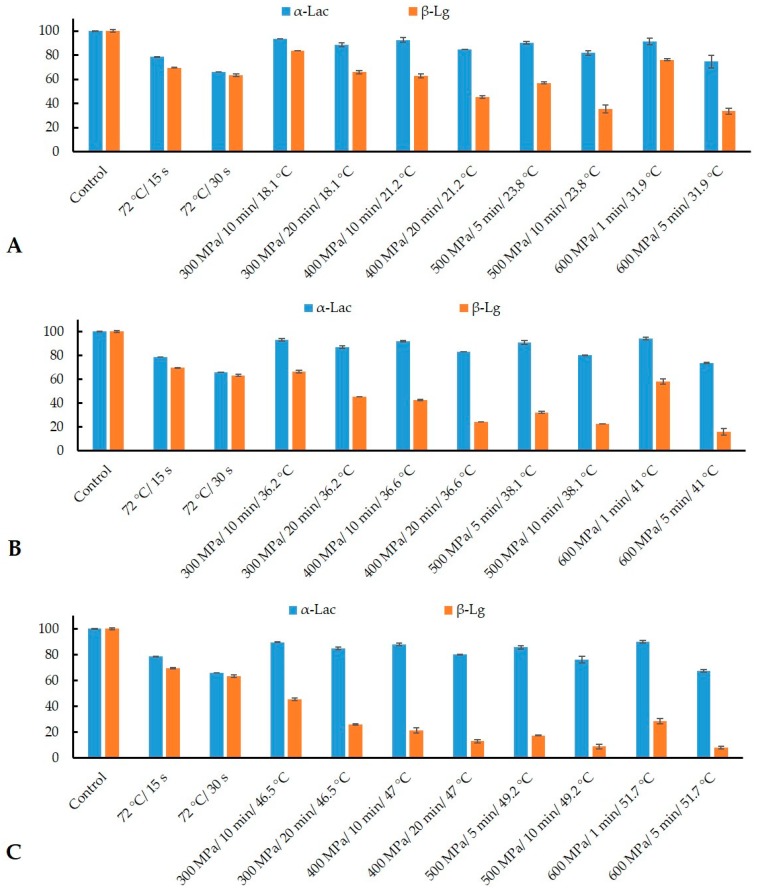
Retention of α-Lac and β-Lg (%) after high-temperate short-time (HTST) pasteurization at 72 °C for 15 and 30 s, and HPP applied at ~20 °C (**A**), ~30 °C (**B**), and ~40 °C(**C**). Error bars represent the standard deviations of duplicates.

In this study, the retention of α-Lac and β-Lg after HTST and HPP was measured by ELISA and compared thereafter (Figure 6). Before treatment, the concentration of α-Lac and β-Lg in reconstituted IMF was 1.04 and 6.2 mg/mL, respectively. Conventional HTST (72 °C for 15 and 30 s) retained 78% and 70% of α-Lac and β-Lg respectively, which corroborates the results reported previously [27,41]. However, in contrast, the degree of denaturation of β-Lg was more pronounced than α-Lac, as observed with HPP combinations of increased pressure, temperature, and time (Figure 6). This trend is in agreement with those obtained from HPP in skim milk [31,32] and in a protein solution [42].

**Figure 7:**
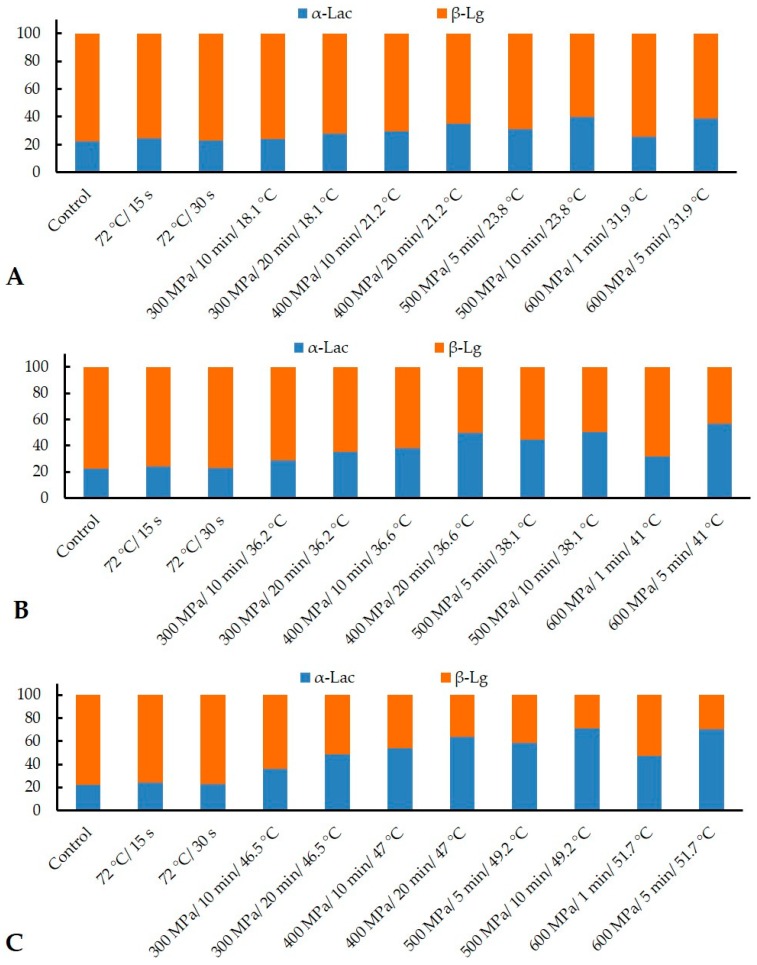
Relative proportions of α-Lac and β-Lg (%) after HTST at 72 °C for 15 and 30 s, and HPP applied at ~20 °C (**A**), ~30 °C (**B**), and ~40 °C (**C**). Total refers to the sum of α-Lac and β-Lg content.

Figure 7 represents the relative proportions (%) of α-Lac and β-Lg derived from Figure 6. The highest ratio of α-Lac to β-Lg (77:23) was achieved from the HPP treatment of 600 MPa at 51.7 °C for 5 min, whereas it was only 24:76 and 23:77 in HTST for 15 and 30 s, respectively (Figure 7). From the results obtained in this work, it is evident that the synergistic effect of HPP at elevated temperature induces a higher ratio of α-Lac to β-Lg, compared to the untreated (22:78) and HTST-treated α-Lac-added reconstituted IMF. The higher baroresistance of α-Lac, compared with β-Lg, is consistent with previous observations in milk [31,32,43]. This difference is considered to be due to the higher number of intramolecular disulfide bonds (4 in α-Lac and 2 in β-Lg) and to the presence of a free sulphydryl group in β-Lg [32,40]. Upon unfolding of β-Lg due to HPP, this free sulphydryl group interacts with proteins containing disulphide bonds (e.g., αs2-casein, k-casein, α-Lac, and β-Lg) through sulphydryl–disulfide interchange reactions resulting in aggregation. Moreover, unfolded α-Lac and β-Lg, which did not interact with other proteins, refold to their native forms on the release of pressure [31]. Therefore, the mechanistic approach of using HPP followed in this work explains the mechanism to achieve a final product with a massive reduction in the β-Lg portion, which subsequently would result in lowering the allergenicity and protein content.

The results found in this work show the potential route to develop an HPP-treated pasteurized RTF hypoallergenic formula because of having a higher ratio of α-Lac to β-Lg. In addition to this, such a formula would ensure the required amino acid balance in the treated product due to the α-Lac supplementation, which may also compensate the lower contribution of heavily denatured β-Lg in the amino acid profile. Thus, this work streamlines the possibility of manufacturing a hypoallergenic and low-protein pasteurized RTF formula. However, further investigation in post-treatment analysis (e.g., bioavailability, digestibility, amino acid profile, etc.) of HPP-treated formula is highly recommended to commercialize this research. Besides, the shorter shelf life at refrigerated conditions of this pasteurized product than that of the sterilized RTF formula would result in slower progress in gaining market. Moreover, HPP is still limited by its batch operation although the recent patent-pending concept of Hiperbaric, the HPP equipment manufacturer, to process liquid foods up to 10,000 L/h before bottling (aseptic packaging) is being considered as a promising innovation to address HPP’s batch operation [44].

## 4. Conclusions

Our results demonstrated the synergistic effect of HPP and heat on the substantial reduction of β-Lg in reconstituted IMF added with α-Lac. Compared to HTST, the HPP treatment at 600 MPa for 5 min applied at 40.4 °C achieved the higher ratio of α-Lac to β-Lg. Overall, the pronounced reduction of β-Lg due to the combined effect of HPP and heat confirms the possibility to manufacture a hypoallergenic and low protein RTF formula, a niche product. Further investigation is necessary to explore the post-treatment effects on physicochemical and rheological properties, along with microbial studies.

## Figures and Tables

**Table 1:** HPP conditions at different temperatures and the corresponding parameters.

Pressure	300 MPa			400 MPa			500 MPa			600 MPa		
Pressure come up time (s)	60	60	60	80	80	80	90	90	90	100	100	100
Initial temperature (°C)	14.8	30.4	41.2	14.7	29.9	40.2	14.3	29.9	40.7	21.2	30.3	40.4
Maximum temperature, *T*_max_(°C)	21.6	38.5	51.2	23.9	40.2	50.8	27.2	42.3	54.6	35.9	45.1	56.4
*T*_eff_ (°C)	18.1	36.2	46.5	21.2	36.6	47	23.8	38.1	49.2	31.9	41	51.7

**Table 2:** Activation energy (*E*_a_) after HPP of 300–600 MPa.

HPP	Activation Energy, *E*_a_ (kJ/mol)	
	α-Lac	β-Lg
300 MPa	8.74	44.8
400 MPa	9.44	51.3
500 MPa	9.65	52.5
600 MPa	12.13	71.6
